# Application of ensemble machine learning approach to assess the factors affecting size and polydispersity index of liposomal nanoparticles

**DOI:** 10.1038/s41598-023-43689-4

**Published:** 2023-10-21

**Authors:** Benyamin Hoseini, Mahmoud Reza Jaafari, Amin Golabpour, Amir Abbas Momtazi-Borojeni, Maryam Karimi, Saeid Eslami

**Affiliations:** 1 Pharmaceutical Research Center, Pharmaceutical Technology Institute, Mashhad University of Medical Sciences, Mashhad, Iran; 2 Nanotechnology Research Center, Pharmaceutical Technology Institute, Mashhad University of Medical Sciences, Mashhad, Iran; 3 Department of Pharmaceutical Nanotechnology, School of Pharmacy, Mashhad University of Medical Sciences, Mashhad, Iran; 4 Department of Health Information Technology, School of Allied Medical Sciences, Shahroud University of Medical Sciences, Shahroud, Iran; 5 Department of Medical Biotechnology, School of Medicine, Neyshabur University of Medical Sciences, Neyshabur, Iran; 6 Healthy Ageing Research Centre, Neyshabur University of Medical Sciences, Neyshabur, Iran; 7 Institute of Human Virology, School of Medicine, University of Maryland, Baltimore, USA; 8 Department of Medical Informatics, Faculty of Medicine, Mashhad University of Medical Sciences, Mashhad, Iran

**Keywords:** Drug delivery, Biotechnology, Computational biology and bioinformatics, Drug discovery

## Abstract

Liposome nanoparticles have emerged as promising drug delivery systems due to their unique properties. Assessing particle size and polydispersity index (PDI) is critical for evaluating the quality of these liposomal nanoparticles. However, optimizing these parameters in a laboratory setting is both costly and time-consuming. This study aimed to apply a machine learning technique to assess the impact of specific factors, including sonication time, extrusion temperature, and compositions, on the size and PDI of liposomal nanoparticles. Liposomal solutions were prepared and subjected to sonication with varying values for these parameters. Two compositions: (A) HSPC:DPPG:Chol:DSPE-mPEG2000 at 55:5:35:5 molar ratio and (B) HSPC:Chol:DSPE-mPEG2000 at 55:40:5 molar ratio, were made using remote loading method. Ensemble learning (EL), a machine learning technique, was employed using the Least-squares boosting (LSBoost) algorithm to accurately model the data. The dataset was randomly split into training and testing sets, with 70% allocated for training. The LSBoost algorithm achieved mean absolute errors of 1.652 and 0.0105 for modeling the size and PDI, respectively. Under conditions where the temperature was set at approximately 60 °C, our EL model predicted a minimum particle size of 116.53 nm for composition (A) with a sonication time of approximately 30 min. Similarly, for composition (B), the model predicted a minimum particle size of 129.97 nm with sonication times of approximately 30 or 55 min. In most instances, a PDI of less than 0.2 was achieved. These results highlight the significant impact of optimizing independent factors on the characteristics of liposomal nanoparticles and demonstrate the potential of EL as a decision support system for identifying the best liposomal formulation. We recommend further studies to explore the effects of other independent factors, such as lipid composition and surfactants, on liposomal nanoparticle characteristics.

## Introduction

Over the past few decades, the field of drug delivery has experienced significant growth, with further innovations anticipated^1–3^. Drug delivery systems describe how pharmaceutical compounds are ‘packaged’—like a nanoparticle—that protects the pharmaceutical compounds from degradation^4^. In recent years, Nano-drug delivery systems (NDDS) have emerged as a promising approach to overcome the challenges associated with drug delivery, which can limit its therapeutic potential^5–10^. NDDSs enable controlled release and targeted delivery of pharmaceutical compounds while reducing non-specific interactions in undesirable tissues^11–14^. The size of nanoparticles affects most phenomena, such as selective targeting of tumors and enhanced oral drug absorption^15,16^. Evidence suggests that smaller particles are more effective in reaching their intended targets^17^. Thus, particles should be controlled for their size and size distribution in NDDSs. Size distribution is described by the polydispersity index (PDI), specifying the uniformity of nanoparticles, which a PDI value of 0.1 to 0.25 indicates a narrow size distribution while a PDI greater than 0.5 refers to a broad distribution^18,19^.

Particle size and PDI are two essential parameters of evaluating a formulation of drug-loaded nanoparticles^20–25^, which depend upon different factors such as compositions, sonication time, and extrusion temperature^24^. To obtain a minimum particle size with a narrow size distribution, these independent factors are routinely manipulated by empirical methods, relying on the iterative TRAIL-and-ERROR approach^20,24^. The substantial drawbacks of this method are the restricted number of experiments and the one-factor-at-a-time approach to doing these experiments^26,27^. Besides, this approach ignores the interactions between the factors affecting a parameter. Accordingly, developing models that can predict the size and PDI of nanoparticles would be very beneficial, as it will save cost by preserving the materials consumed during the optimization of the formulations.

Design of Experiments (DoE), exemplified by Box-Behnken designs, serves as the primary experimental framework within response surface methodology (RSM)^7^. Artificial Neural Networks (ANNs) and RSM represent two distinct techniques harnessed for the optimization and prediction of nanoparticle characteristics^26,28–40^. ANNs are characterized by their data-driven approach, proficiency in handling non-linearity, and widespread application in machine learning contexts^37,41^. Conversely, RSM adopts a statistical paradigm meticulously tailored for modeling and optimizing processes, relying on empirical data and mathematical equations^7,29^. The selection between these methodologies hinges upon factors such as the specific problem domain, data availability, and the necessity for interoperability. Recent research has notably illustrated the superiority of ANNs over RSM in optimizing nanoparticle properties^29,32,42,43^. Nonetheless, ANNs are not without limitations, notably their propensity to converge on local optima and their confinement within a limited factor space^41,44^. In scenarios necessitating profound insights into intricate phenomena, an alternative approach becomes imperative. Ensemble learning (EL) modeling is one way to overcome these barriers, as it applies algorithms to learn from data and predict outcomes^45–48^.

An EL approach is a generalized meta method of machine learning, seeking to predict better by combining predictions from multiple learning models^49^. This method is a vital interpretation of experimental outcome, hence may provide better insight into the optimal formulation parameters^50^. In this sense, models for particle size and PDI linked to EL may be useful for assessing relationships between inputs and outputs. Although liposomal drug-loaded nanoparticles have been widely studied in the field of drug delivery^13,24,40,51,52^, to our knowledge, no study has addressed the EL technique as a tool for the optimization of input factors to experimentally obtain the minimum responses in liposomal particle size and PDI.

Liposomes are small, spherical artificial vesicles that contain at least one lipid bilayer^53^. The hydrophobicity and/or hydrophilicity, biocompatibility, particle size, high loading capacity, controlled-release properties of loaded drugs and many other characteristics of liposomes make them ideal drug delivery nanoparticle for pharmaceuticals and nutrients^54–57^. Liposomes have been recently used for the delivery of curcumin^24,30,51^. Curcumin is the main active constituent of turmeric isolated from the plant Curcuma longa^58^. Curcumin has a broad spectrum of biological effects including anti-inflammatory, antioxidant, antiangiogenic^59^ and the potential anticancer activities to treat various types of cancers^60–62^. However, short half-life, fast metabolism, chemical instability, as well as poor water solubility and bioavailability are important barriers of curcumin, restricting its clinical applications^63^.

Proper formulation and processing studies can identify the composition and manufacturing methodologies to make liposomes of the appropriate particle size with excellent stability^64^. Thus, stable liposomes that can deliver loaded curcumin with excellent pharmacodynamics and pharmacokinetics are an urgent need in the field of lipid drug delivery. In addition, particle size and PDI can influence how much of the loaded curcumin will be released and absorbed upon reaching the target site, directly affecting its pharmacological characteristics. Applying EL to optimize the curcumin-loaded liposomal nanoparticles, as a case study, might shed some light on the capabilities of this technique in the field of drug delivery. Accordingly, the study aimed to assess the impact of specific factors, including sonication time, extrusion temperature, and molar ratios, on the size and PDI of liposomal nanoparticles using EL.

## Material and methods

### Materials

Curcumin (Sami Labs Limited, Bengaluru, Karnataka, India) and Caelyx^®^ (Janssen Pharmaceuticals, Inc., a subsidiary of Johnson & Johnson; Behestan Darou Company, Tehran, Iran). HSPC, DPPG, Chol and DSPE-mPEG2000 were purchased from Avanti Polar Lipid (Alabaster, USA). Dimethyl sulfoxide (DMSO), ethanol and chloroform (Merck, Darmstadt, Germany). All chemicals and solvents used were of analytical grade.

The preparation of liposomes and drug loading encompassed a preliminary screening study in which two specific compositions were identified for further investigation. These compositions were designated as (A) HSPC:DPPG:Chol:DSPE-mPEG2000 with a molar ratio of 55:5:35:5, and (B) HSPC:Chol:DSPE-mPEG2000 with a molar ratio of 55:40:5. These compositions exhibited exceptional performance in terms of high encapsulation efficiency (EE%) and were created using the remote loading method.

To initiate the process, lipids dissolved in chloroform were combined in a round-bottom flask, forming a lipid film. Subsequently, the solvent was evaporated under vacuum conditions using a rotary evaporator (Heidolph, Germany). Lyophilization, performed using a specialized lyophilizer (VD-800F, Taitech, Japan), was then conducted for 2 h to eliminate any residual solvent traces. The resultant lipid film was subsequently hydrated in pre-warmed absolute ethyl alcohol within a hydration buffer (Phosphate-buffered saline (PBS) at 10% (v/v)). The thin film was initially dissolved in absolute ethanol, followed by the addition of the pre-heated hydration buffer (at about Tm temperature). This mixture was vortexed using a vortex shaker to ensure proper dispersion of the lipid blend within the buffer, resulting in the formation of large multilamellar vesicles (MLVs).

To further refine the liposomal structure, the MLVs underwent a 30-min sonication process within a bath sonicator (Bandelin Electronics, Germany) set at a temperature of 55 °C. Subsequently, a series of steps were undertaken to generate small unilamellar vesicles (SUVs). This involved the sequential extrusion of liposomes through a thermobarrel extruder (Avestin, Canada), employing a series of polycarbonate filters (Whatman, Maidstone, Kent, UK) with diminishing pore sizes: 400 nm, 200 nm, 100 nm, and finally 50 nm. The formulations underwent 11 rounds of extrusion through each filter.

The determination of the temperature for formulation preparation was guided by the phase transition temperature (Tm) of the phospholipids within each liposomal formulation. Given the predominant presence of HSPC in our compositions, constituting 55% of the molar ratio, the Tm was ascertained based on the phase transition temperature of HSPC.

To encapsulate curcumin into liposomes, the solvent-assisted active loading technology (SALT) involving DMSO was employed^65^. This novel technique incorporates a hydrophobic drug into the liposomal core, as a very small quantity of DMSO rapidly and effectively disrupts the assembly of liposomes, then the hydrophobic drug enters the liposomal core, and after that, the liposomes reform again. This method has been previously proven by Tang et al.^65^, to not significantly affect the liposome structure, allowing hydrophobic drugs to be incorporated into the liposomal core. Following this method, a curcumin solution was prepared by dissolving 2 mg/ml of curcumin in DMSO, ensuring complete dissolution through vigorous vortexing. Subsequently, the prepared curcumin solution was added to the liposomes at 65 °C for 10 min, with a DMSO concentration of as low as 5% at this step. In order to remove the free curcumin and DMSO, liposomal curcumin was dialyzed (12–14 kDa MWCO) against PBS buffer at a ratio of at least 1 to 100. All the final prepared liposomal formulations were sterilized through filtration using 0.22 µm syringe filter^66^.

### Nanoliposomal characterization

The particle size and PDI were measured by Dynamic Light Scattering instrument (Nano-ZS; Malvern, UK)^67^. The amounts of phospholipids were determined by the Bartlett phosphate assay method^68^. Also, the morphological feature of liposome was evaluated using transmission electron microscopy (TEM) via negative staining^69^. The sample was prepared for TEM photography as follows: first liposome was diluted (1:40 of liposome to dialysis buffer (PBS)) and 20 µL of sample was dropped onto a carbon-coated copper grid. After 1 min, the excess liposome was removed by filter paper. Then 20 µL filtered uranyl acetate (2% w/v) was dropped onto grid. After drying, the samples were photographed with a LEO 912 TEM at an accelerating voltage of 80 kV (Jena, Germany).

To assess the stability of the liposomal formulations, a comprehensive liposome stability assessment was conducted. The investigation covered a storage duration of 24 weeks under controlled conditions of 4 °C and 25 °C. Key parameters, including size distribution, zeta potential, PDI, and EE%, were rigorously analyzed at various intervals (0, 4, 12, and 24 weeks post-preparation). The detailed procedure and outcomes of this assessment are provided in the Supplementary file.

### Ensemble learning modeling method

EL is a modeling method where multiple prediction models are combined to make joint decisions, taking advantage of the strengths of each individual model^49^. Each prediction model has its own set of advantages and disadvantages, as well as specific suitability for different data domains and volumes. By combining these predictions, the accuracy of the overall prediction is improved, compensating for any individual model’s limitations. To make the prediction result better, two conditions should be addressed for EL: (1) There must be a difference between each prediction model; (2) The accuracy of each prediction model should be > 0.5. Theoretically, the prediction will have the better accuracy. If both conditions are met and the weak models are combined.

Least-squares boosting (LSBoost)^70^ is a sophisticated machine learning algorithm frequently employed in EL methodologies. At the core of this algorithm are individual prediction models known as ‘weak learners’ or ‘trees’. These weak learners are essentially like small prediction modules that, on their own, might not be particularly accurate or robust. However, they are systematically combined to create a more powerful and accurate predictive model^71,72^. At every iteration step, the ensemble fits in a fresh learner, as puzzle pieces that, when assembled correctly, form a complete picture.

In the context of LSBoost, the algorithm works by involving hundreds of these weak learners, each of which is designed to make a prediction. Through a series of iterative steps, the algorithm aims to improve the overall predictive accuracy. It does this by focusing on correcting the errors made by the previous weak learners in subsequent iterations. This correction process gradually transforms the collection of weak learners into a ‘strong learner’, which is a much more accurate and reliable predictive model. Thus, LSBoost employs a multitude of weak learners to collectively build a strong and precise predictive model through iterative error correction. This approach leverages the strengths of individual models to achieve a more robust and effective overall prediction.

The mean square error (MSE) is utilized as a measurement to assess the variance between the actual outcomes (*Y*_i_) and the predicted outcomes (*f*(*X*_i_)) for each observation and is estimated using Eq. (1) as follows^72^:

1 $$MSE= L\left(Y, {f(X}_{i})\right)= \sum_{i=1}^{K}{\left({Y}_{i}+{f(X}_{i})\right)}^{2},$$

where *Y*_*i*_,* f*(*X*_*i*_), and *K* indicate the actual output, the predicted output generated by the model, and the number of samples, respectively. In this study, outputs were particle size and PDI, and *K* was the number of experiments.

Owing to the hypothetical bias and variance issues, the fitted model and the resulting predicted outcome may severely suffer from underfitting or overfitting problems, leading to a high error between the targeted response and the estimated variables. In order to address such drawbacks, the inconsistency of *f*(X_i_) in Eq. (1) needs to be placed under control by employing the bagging or LSBoost algorithms^72^. Bagging, also known as Bootstrap aggregating^73^, is an EL technique that involves creating multiple models using different subsets of the training data, obtained through random sampling with replacement. These models are then aggregated to reduce variance and improve overall prediction performance. It helps to reduce the risk of overfitting by introducing diversity into the models.

This study addressed the LSBoost algorithm for the prediction of particle size and PDI. This choice was motivated by the ensemble methods modeling capabilities of nonlinear and non-stationary problems. These methods have several advantages, i.e. flexible input, ability to indirectly identify dynamic non-linear interactions between dependent and independent predictors, ability to identify all potential interactions between predictors and have demonstrated high performance in solving medical prediction challenges.

Additionally, the mean absolute error (MAE), which quantifies the average absolute difference between the predicted values ($\hat{y}_{i}$) generated by the model and the actual values (y_i_), is employed as the primary cost function in this study. Equation (2)^67^ defines the MAE as the below^74^:

2 $$MAE =1/\mathrm{n }\sum_{i=1}^{n}\left|{y}_{i}- \widehat{{y}_{i}}\right|,$$

where *y*_*i*_*,* $\hat{y}_{i}$ and *n* indicate the actual output, the predicted output generated by the model, and the number of samples, respectively. In this study, outputs were particle size and PDI, and *n* was the number of experiments.

The ensemble methods required training of the models to find the optimal set of the parameters. Thus, the data was randomly divided such that 70% of the data were used for training of the models and rest of them (30%) were reserved for testing. This procedure ensured that the results were not prone to overfit and would be transferable to the similar settings. Table 1 shows these training and testing datasets used in our EL modeling. The decision to report the particle size and PDI separately in Table 1 is driven by the fact that each of these parameters is individually modeled by the EL algorithm. Even though the input data for both parameters are the same, they are treated as separate outcomes in the modeling process. As a result, during the random allocation of data into training and testing subsets, a data record for one parameter might end up in the training set, while the same record for the other parameter might be placed in the testing set. This distinction occurs only within the modeling framework, and it's important to note that during actual experimental measurements, both particle size and PDI are naturally measured simultaneously for a given set of conditions (see Table S1 in supplementary file).

**Table 1:** The training and testing datasets used in machine learning modeling.

Composition	Time (min)	Temp (°C)	Measured size (nm)	Predicted size (nm)	Composition	Time (min)	Temp (°C)	Measured PDI	Predicted PDI
Training dataset									
A	15	27	139.60	138.71653	B	60	27	0.284000	0.290500
B	45	55	136.70	138.68319	A	30	45	0.111000	0.105721
B	45	27	147.10	146.44182	B	30	45	0.171000	0.178962
B	15	27	143.90	144.49479	A	15	65	0.020000	0.027361
B	15	55	135.70	134.25234	A	15	27	0.136000	0.133870
A	30	45	118.90	118.03337	B	45	27	0.297000	0.290178
B	15	45	139.60	141.11013	A	45	27	0.134000	0.148558
A	45	27	164.80	169.07407	B	15	65	0.164000	0.156373
A	15	65	121.40	121.59488	A	60	27	0.219000	0.208004
A	60	27	181.80	178.03320	B	60	45	0.181000	0.177430
B	60	27	154.80	155.05217	A	60	45	0.161000	0.163312
A	45	55	157.90	154.43624	B	30	27	0.265000	0.275490
A	30	65	114.80	116.52785	B	30	55	0.141000	0.156373
B	30	45	137.80	136.82434	B	15	27	0.288000	0.275490
B	45	45	143.70	141.46544	A	60	65	0.102000	0.108920
A	60	45	159.20	160.82535	A	30	55	0.041000	0.027361
A	60	65	144.60	145.75851	B	45	55	0.218000	0.209098
B	60	45	149.10	150.07579					
Testing dataset									
A	30	27	131.80	133.64949	A	45	45	0.103000	0.120408
A	15	45	128.70	123.10041	B	45	45	0.197000	0.193650
A	45	45	152.10	151.86622	A	30	65	0.022000	0.027361
B	30	27	140.20	140.20899	B	30	65	0.133000	0.156373
A	30	55	117.30	116.52785	B	15	55	0.169000	0.156373
B	15	65	132.40	134.25234	A	45	55	0.086000	0.080087
B	30	65	129.40	129.96655	A	30	27	0.139000	0.133870
B	30	55	132.30	129.96655					

Compositions: (A) HSPC:DPPG:Chol:DSPE-mPEG2000 at 55:5:35:5 molar ratio and (B) HSPC:Chol:DSPE-mPEG2000 at 55:40:5 molar ratio. Time (min) = sonication time (minutes), Temp (°C)= extrusion temperature (°C), PDI= polydispersity index.

LSBoost algorithm was implemented using Matlab R2020a (The Mathworks Inc., Natick, Massachusetts). The programming codes are presented in Supplementary file. The parameters were optimized regarding performance and transferability using the training/validation split of the data. LSBoost was optimized for finding the best number of trees, learning cycles, and leaf size. The following search space for the parameter optimization was: Type: 'regression'; Method: 'LSBoost'; LearnerTemplates: 'Tree'; NLearn: 100; LearnRate: 0.2000.

### Inputs and output variables

As it mentioned before, particle size and PDI were endpoints of interest in this study. We modeled the effect of three factors (compositions, sonication time, and extrusion temperature) on these parameters. All experimental conditions set for input factors along with actual values measured for particle size and PDI are outlined in Table S1 (see Supplementary file). The Shapiro–Wilk test using IBM SPSS Statistics (version 20.0, IBM Corp., Armonk, NY, USA) was applied to examine the normality of the data.

### Response surfaces

The 3D plots were depicted to demonstrate the relationships between input factors and their effect on the particle size and PDI (i.e. outputs). These plots allow for the effective representation of how two input factors influence the endpoint of interest at a given time. To comprehensively depict the combined effect of all three input factors on particle size and PDI, we employed a specific approach. Since one of the factors, composition, was binary in our study, we chose to illustrate the impact of the remaining two factors on particle size and PDI separately for each composition. This approach allowed us to capture the nuanced effects of the input factors on the desired outcomes for both compositions.

### Ethics approval and consent to participate

The study received approval from the Mashhad University of Medical Sciences Ethics Committee. No human or animal experiments were conducted as part of this study.

## Results

After modeling the normally distributed data, the best predictive models yielded MAEs of 1.6520 for particle size and 0.010452 for PDI on the testing datasets. Table 2 presents the parameters evaluated for both the trained and test models, indicating the robust predictive capability of the trained models. The measured and predicted values for particle size and PDI are displayed in Table 1, showcasing a remarkably close match between the model predictions and the measured values. This minimal deviation underscores the validity of the ensemble models.

**Table 2:** Optimization and prediction capability of ensemble learning for particle size and PDI.

Parameters	Particle size		PDI	
	Trained model	Test model	Trained model	Test model
MSE	3.8361	5.5780	8.6112e-05	1.5858e-04
MAE	**1.5885**	*1.6520*	**0.0084089**	*0.010452*
PSNR	42.2919	40.6660	40.6494	37.9976
Rvalue	0.9998	0.9997	0.9976	0.9910
RMSE	1.9586	2.3618	0.0093	0.0126
NRMSE	0.0292	0.0679	0.0335	0.072

*PDI* polydispersity index, *MSE* mean squared error, *MAE* mean absolute error, *PSNR* peak signal-to-noise ratio definition, *RMSE* root mean square error, *NRMSE* normalized root mean square error.

Significant values are in bold and italic.

**Figure 1:**
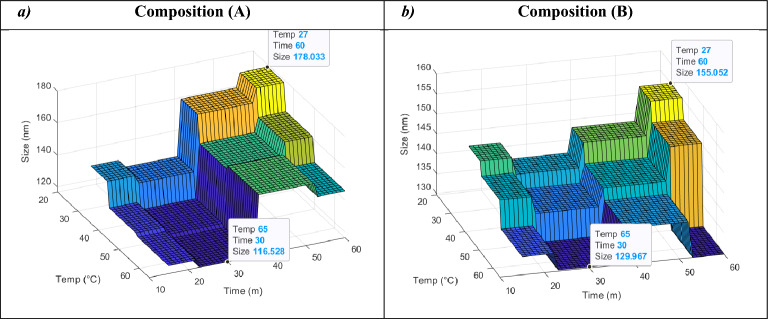
Predicted 3D response surface plots of size (nm) as a function of temp (°C) and time (m) for two different liposomal compositions: (A) HSPC:DPPG:Chol:DSPE-mPEG2000 at 55:5:35:5 molar ratio (**a**) and (B) HSPC:Chol:DSPE-mPEG2000 at 55:40:5 molar ratio (**b**). The figure illustrates the variations in particle size in relation to sonication time and extrusion temperature. The compositions’ significance is reflected in their distinct responses. Size (nm) represents particle size (nm), Time (m) signifies sonication time (minutes), and Temp (°C) denotes extrusion temperature (°C).

**Figure 2:**
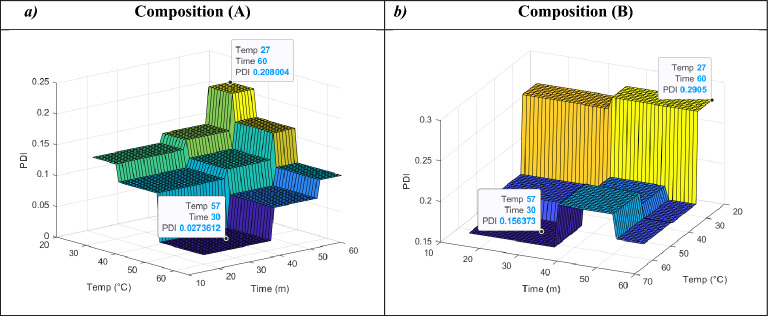
Predicted 3D response surface plots of PDI as a function of temp (°C) and time (m) for two different liposomal compositions: (A) HSPC:DPPG:Chol:DSPE-mPEG2000 at 55:5:35:5 molar ratio (**a**) and (B) HSPC:Chol:DSPE-mPEG2000 at 55:40:5 molar ratio (**b**). The figure illustrates the variations in PDI in relation to sonication time and extrusion temperature. The compositions’ significance is reflected in their distinct responses. Size (nm) represents particle size (nm), Time (m) signifies sonication time (minutes), and Temp (°C) denotes extrusion temperature (°C).

The response surface 3D plots illustrate the relationships between input factors (compositions, sonication time, and extrusion temperature) and the responses/outputs (particle size (Fig. 1) and PDI (Fig. 2)).

For composition (A), the minimum predicted values for particle size and PDI were determined to be 116.53 nm and 0.027, respectively, indicating that composition (A) is associated with both smaller particle size and a lower PDI. Conversely, composition (B) exhibited minimum predicted values of 129.97 nm for particle size and 0.156 for PDI.

In general, the plots illustrating the impact of sonication time and extrusion temperature on particle size for both compositions exhibited consistent trends (refer to Fig. 1). As the temperature increased from 27 °C to 65 °C, a notable decrease in particle size was observed, reaching a minimum at approximately 60 °C. Conversely, particle size exhibited an upward-downward pattern as sonication time increased from 15 to 60 min. For composition (A), the EL model predicted a minimum particle size of 116.53 nm at approximately 30 min of sonication when the temperature was set at around 60 °C (see Fig. 1a). In composition (B), the model projected a minimum particle size of 129.97 nm at sonication times of approximately 30 or 55 min (see Fig. 1b).

PDI responses were modeled as functions of sonication time and extrusion temperature for two distinct compositions, as illustrated in Fig. 2. In both compositions, the minimum PDI values were attained at specific conditions: approximately 15–37 min of sonication time and around 50–65 °C for temperature. These conditions yielded predicted PDIs of 0.027 and 0.156 for compositions (A) and (B), respectively. Notably, in composition (A), PDI exhibited a decreasing trend as temperature increased and sonication time decreased. In contrast, for composition (B), the pattern varied slightly, resulting in a PDI of 0.178 under conditions of 55–60 min of sonication and 37–65 °C of temperature. It's worth mentioning that, in most instances, PDIs less than 0.2 were achieved.

## Discussion

The appropriateness of liposomal formulations for a specific route of drug administration hinges upon the physicochemical properties of the liposomes, encompassing parameters such as particle size, PDI, and surface charge^75^. The meticulous control of these factors holds significant promise in enhancing the biodistribution and pharmacokinetics of liposomal formulations, thereby contributing to improved clinical outcomes of drug therapies^27,75–77^. Particle size and size distribution of liposomes are pivotal determinants, significantly influencing attributes such as EE, stability, drug release kinetics, cellular uptake, and biodistribution^78^. In this study, we systematically employed the EL technique to assess the impact of three independent factors, namely, compositions, sonication time, and extrusion temperature, on the particle size and PDI of nanoliposomes. Our findings strongly corroborate the established relationships between these independent factors and the parameters under assessment, in alignment with prior research endeavors^20,30,79,80^.

Liposomes exhibit a diverse range of sizes, ranging from very small (0.025 μm) to large (2.5 μm) vesicles, and may possess single or bilayer membranes^53^. By employing EL-designed models, the study predicted the minimum liposome sizes to be 116.52 nm and 129.97 nm for two distinct compositions. These predictions highlight that composition (A) is associated with a smaller particle size and lower PDI, indicating a more uniform and potentially more stable formulation of liposomal nanoparticles. Conversely, composition (B) demonstrates a slightly larger particle size and higher PDI, suggesting a less uniform distribution of nanoparticles. These predictive results offer valuable insights for optimizing the formulation process and selecting the appropriate composition to achieve the suitable particle size and PDI properties.

Cholesterol plays a pivotal role as a primary component in our liposomal compositions. As observed by Shaker et al.^80^, there exists a direct correlation between the concentration of cholesterol and the size of liposomes. In our study, larger liposomes, which are generally considered undesirable in liposomal compositions, were observed in composition (B) with a higher cholesterol concentration. It’s worth noting that while cholesterol may influence liposome properties, the role of other components, particularly DPPG, should not be overlooked. To gain a more comprehensive understanding of the interplay between these constituents, we suggest assessing different compositions such as HSPC:Chol:DPPG at 60:35:5 in future research. This will enable a deeper exploration of the contributions of cholesterol and DPPG to liposomal characteristics and aid in refining our liposomal formulations.

Although the augmentation of cholesterol may correlate with an enlargement in liposome size, the inclusion of cholesterol yields several advantageous outcomes within the composition. It substantially heightens the stability of the liposomes by bolstering their resistance to aggregation, diminishing bilayer permeability, and fostering more efficient packing of phospholipids. Consequently, cholesterol contributes to increased rigidity of the lipid bilayer and reduced drug leakage, thereby augmenting the overall integrity of the liposomes^80–84^.

Moreover, it is crucial to acknowledge that particle size also has a significant impact on the EE of liposomal compositions, making it an important parameter to consider when selecting the optimal formulation^78^. Researchers should carefully evaluate and balance all of the aforementioned parameters to develop a formulation that meets their specific requirements. In this context, EL can serve as a valuable decision-support system. While EL provides insights and support, researchers should ultimately rely on their expertise and knowledge to make informed decisions in conjunction with the guidance provided by EL.

PDI serves as a crucial indicator of colloidal dispersion homogeneity, with values exceeding 0.7 typically denoting a broad size distribution^85^. Modeling by EL technique yielded the PDIs lower than 0.16 for both compositions, signifying that these specific compositions did not exert a substantial influence on PDI within the confines of our study. While researchers possess the flexibility to select either composition based on other pertinent parameters, it is imperative to acknowledge that our study refrained from assessing an extensive spectrum of compositions and/or molar ratios.

As a consequence, it is not tenable to assert that compositions and/or molar ratios lack a considerable impact on PDI within our study's boundaries. Thus, further inquiries involving a more comprehensive exploration of compositions and/or molar ratios are imperative to comprehensively discern the implications on PDI. Our study revealed that despite both liposomal formulations yielding homogeneous populations, the EL analysis illuminated the susceptibility of PDI to influences such as sonication time and temperature.

Sonication is a simple approach for reducing the size of liposomes^79^. We modeled the size of liposomes as a function of sonication time using EL technique, showing the strong association between time and size in both compositions. By increasing time, liposome size first decreases until about a specific time and then rises again that is in line with the previous studies^86–88^. Yamaguchi et al. reported that the high-intensity focused ultrasound could affect the size of the liposomes^89^. Paclitaxel-liposomes and liposomes formulated for enhanced thrombolysis have also shown a similar trend^90,91^.

One of the methods commonly used to prepare liposomes is extrusion, which involves passing a lipid mixture through a series of filters with decreasing pore sizes^24^. The extrusion temperature can have a significant impact on the size of the resulting liposomes. Higher temperatures may result in larger liposomes due to increased lipid mobility and fusion events during the extrusion process. In our study, the size reduced substantially when the temperature increased to about 37 °C in the composition (A) and, in line with another study^92^, revealed that the increment of temperature above 37 °C does not affect the size and size distribution of the liposomes, especially at 30 min sonication time. In composition (B), a substantial reduction in size and size distribution appeared when the temperature increased above 50 °C. Thus, controlling the extrusion temperature is critical to achieve the desired liposome size and optimizing their properties for various applications.

Machine learning techniques, particularly ANNs, have been successfully used to optimize the formulation of drug-loaded liposomes^30,32,93,94^. These models have proven to be more accurate than traditional linear regression models in predicting liposome properties^93,94^. However, using an ensemble instead of a single model has several advantages, including better prediction accuracy and reduced prediction dispersion^49^. Although the EL technique has been applied in pharmaceutical sciences before^95,96^, this study is the first to use it to optimize liposomal nanoparticles. The study produced MAEs of 1.6520 and 0.010452 for size and PDI, respectively, demonstrating the significant potential of this technique in modeling the complex interactions involved in the drug delivery process.

To the best of our knowledge, this study represents the pioneering application of ensemble learning techniques for the evaluation of factors influencing particle size and PDI in nanoliposomal formulations. Although our investigation successfully attained a favorable particle size and PDI by employing the HSPC:DPPG:Chol:DSPE-mPEG2000 lipid composition at 55:5:35:5 molar ratio, coupled with an impressive EE of 89%, it is crucial to acknowledge that our deliberate selection of two specific lipid compositions during the preliminary screening phase might have inadvertently limited the exploration of alternative lipid compositions that could potentially yield advantageous particle size and PDI outcomes. Furthermore, it is noteworthy that our study deviated from the conventional expectations associated with extrusion methods, which typically yield particles within the 50 to 60 nm range. In contrast, our study resulted in nanoliposomes with a size of 116 nm. However, despite exceeding the conventional size range, this deliberate choice was made with precision to align with our final objective of developing curcumin-loaded liposomes. This particle size was meticulously selected to optimize curcumin delivery to the tumor site, as particle sizes around 116 nm are known to be suitable for tumor accumulation based on the enhanced permeability and retention effect^97^.

## Conclusion

The study provides useful insights into the factors affecting the size and PDI of liposomal nanoparticles. The results indicate that the optimization of the independent factors can significantly affect the characteristics of liposomal nanoparticles, and EL can be used as a decision support system for determining the best liposomal formulation. Further studies are recommended to investigate the effect of other independent factors, such as lipid composition and surfactants, on the characteristics of liposomal nanoparticles.

### Supplementary Information

Supplementary Information.

## Data Availability

The data analyzed in this study is presented in the paper and Matlab programming codes are available in Supplementary file.

## Abbreviations

HSPC: Hydrogenated soy phosphatidylcholine
DPPG: Dipalmitoyl-phosphatidylglycerol
Chol: Cholesterol
DSPE-mPEG2000: Distearoyl-glycero-3-phosphoethanolamine- methoxypolyethylene glycol2000
