# Supplementary material for: Application of ensemble machine learning approach to assess the factors affecting size and polydispersity index of liposomal nanoparticles
Source: Sci Rep. 2023 Oct 21;13:18012. doi: 10.1038/s41598-023-43689-4 (PMC10590434; doi:10.1038/s41598-023-43689-4)
Supplement: Supplementary file 1 — Supplementary Information. [file 41598_2023_43689_MOESM1_ESM.docx]

**Liposome Stability Assessment**

The physical stability of both liposomal formulations (referred to as Composition (A) and Composition (B)) was systematically investigated over a storage period of 24 weeks under controlled conditions of 4°C and 25°C. The evaluation encompassed critical parameters such as appearance, size distribution, zeta potential, polydispersity index (PDI), and encapsulation efficiency (EE%). To conduct this assessment, samples were collected at various time points (0, 4, 12, and 24 weeks’ post-preparation) from each liposomal formulation. The assessment aimed to gauge the stability of the liposomes with regards to their size, EE, visual appearance, propensity for curcumin leakage, and the tendency to form sedimentation.

Furthermore, the curcumin leakage ratio from each liposomal formulation was quantified, comparing the EE% of curcumin after 6 months of storage to its initial EE% at the commencement of the study (time 0).

The stability analysis results for both liposomal compositions under storage conditions revealed: At 4°C, both zeta potential and average diameter exhibited marginal changes, reflecting a relatively stable state. The visual characteristics of the liposomes remained unaltered during the storage period at this temperature.

However, storage at 25°C over a period of 6 months demonstrated notable variations. The average diameter of Composition (A) and Composition (B) increased from 114.8 ± 4.9 nm to 182.4 ± 1.8 nm and 129.4 ± 2.05 nm to 210.9 ± 4.4 nm, respectively. Curcumin leakage was more pronounced at 25°C, leading to a decrease in EE% from 89% to 51% for Composition (A) and from 82% to 32%for Composition (B), corresponding to curcumin quantities of 1.6 mg to 1.0 mg and 1.7 mg to 0.7 mg, respectively.

Furthermore, the size of Composition (A) expanded by approximately 1.5-fold (from 114.8 to 182.4 nm) during the storage at 25°C, while Composition (B) underwent a size increase of about 1.6-fold (from 129.4 to 210.9 nm). Additionally, the visual appearance of Composition (A) remained unchanged, whereas Composition (B) transitioned from transparent yellow to opaque yellow over the course of 24 weeks at 25°C, accompanied by signs of sedimentation. The calculation of EE% is illustrated through Formula 1.

Entrapment efficiency of curcumin (EE%) = (curcumin in liposomes (mg)/curcumin initially added (mg)) 🞨 100 (Formula 1)

The surface charge, as indicated by the zeta potential, serves as a crucial indicator of the strength of electrostatic repulsive forces among nanoparticles. Several factors influence this parameter, including lipid concentration, phospholipid composition, and the presence of incorporated drugs. Notably, in our study, both compositions exhibited predominantly negative zeta potential values. This characteristic plays a pivotal role in enhancing the stability of these liposomal formulations by mitigating the propensity for liposome aggregation, a trend that aligns with findings from analogous investigations [1].

Both liposomal compositions under examination primarily consist of cholesterol, DSPE-mPEG2000, and HSPC. Since HSPC typically exhibits a nearly neutral zeta potential, the negatively charged nature of these liposomes can be largely attributed to the presence of DSPE-mPEG2000 and other negatively charged phospholipids, such as DPPG. It's worth noting that the divergence in zeta potential values between composition (A) and composition (B) may be attributed to the incorporation of DPPG within the composition (A).

**Table S1.** The experimental conditions and actual measured values for particle size and PDI

| **Composition** | **Sonication time (min)** | **Temperature (°C)** | **Particle size (nm)** | **PDI** |
| --- | --- | --- | --- | --- |
| A | 15 | 65 | 121.4 | 0.02 |
| A | 30 | 65 | 114.8 | 0.022 |
| A | 30 | 55 | 117.3 | 0.041 |
| A | 45 | 55 | 157.9 | 0.086 |
| A | 60 | 65 | 144.6 | 0.102 |
| A | 45 | 45 | 152.1 | 0.103 |
| A | 30 | 45 | 118.9 | 0.111 |
| B | 30 | 65 | 129.4 | 0.133 |
| A | 45 | 27 | 164.8 | 0.134 |
| A | 15 | 27 | 139.6 | 0.136 |
| A | 30 | 27 | 131.8 | 0.139 |
| B | 30 | 55 | 132.3 | 0.141 |
| A | 60 | 45 | 159.2 | 0.161 |
| B | 15 | 65 | 132.4 | 0.164 |
| B | 15 | 55 | 135.7 | 0.169 |
| B | 30 | 45 | 137.8 | 0.171 |
| B | 60 | 45 | 149.1 | 0.181 |
| B | 45 | 45 | 143.7 | 0.197 |
| B | 45 | 55 | 136.7 | 0.218 |
| A | 60 | 27 | 181.8 | 0.219 |
| B | 30 | 27 | 140.2 | 0.265 |
| B | 60 | 27 | 154.8 | 0.284 |
| B | 15 | 27 | 143.9 | 0.288 |
| B | 45 | 27 | 147.1 | 0.297 |
| A | 15 | 45 | 128.7 | NA |
| B | 15 | 45 | 139.6 | NA |
| **Compositions**: (A) HSPC:DPPG:Chol:DSPE-mPEG2000 at 55:5:35:5 molar ratio and (B) HSPC:Chol:DSPE-mPEG2000 at 55:40:5 molar ratio | | | | |

**Table S1** demonstrates all experimental conditions set for input factors (Compositions, Sonication time, and Extruder Temperature) along with actual values measured for particle size and PDI, which reveals during actual experimental measurements, both particle size and PDI were naturally measured simultaneously for a given set of conditions.

Matlab programming codes,

1. The Matlab code for LSBoost ensemble learning algorithm to model particle size

while(1)

clc, clear, close all;

%warning('off','all')

%warning

load('data10.mat')

n=randperm(size(in,1));

in=in(n,:);

out=out(n);

in_training=in(1:round(size(in,1)*0.7),:);

out_training=out(1:round(size(in,1)*0.7),:);

in_test=in(round(size(in,1)*0.7)+1:end,:);

out_test=out(round(size(in,1)*0.7)+1:end,:);

t = templateTree('Surrogate','On');

%load('new_data_size10.mat')

bc=fitrensemble(in_training,out_training,'Method','LSBoost');

%bc=fitrensemble(in_training,out_training,'AdaBoostM1');

%bc=fitrensemble(in_training,out_training,'OptimizeHyperparameters','auto','Learners',t, ...

% 'HyperparameterOptimizationOptions',struct('AcquisitionFunctionName','expected-improvement-plus'))

pre_train=bc.predict(in_training);

pre_tast=bc.predict(in_test);

MAE_Training=mean(abs(out_training-pre_train));

MSE_Training=mean(abs(out_training-pre_train).^2);

MAE_Test=mean(abs(out_test-pre_tast));

MSE_Test=mean(abs(out_test-pre_tast).^2);

disp('--------------------------------');

disp(['MAE Training = ',num2str(MAE_Training)])

disp(['MAE Test = ',num2str(MAE_Test)])

disp('-------------Training-------------------');

p_training=CalcPerf(out_training,bc.predict(in_training))

disp('-------------Test-------------------');

p_test=CalcPerf(out_test,bc.predict(in_test))

if(MAE_Training<MAE_Test && MAE_Test<1.7)

break

end

end

1. The Matlab code for LSBoost ensemble learning algorithm to model PDI

while(1)

clc, clear, close all;

%warning('off','all')

%warning

load('data11.mat')

n=randperm(size(in,1));

in=in(n,:);

out=out(n);

in_training=in(1:round(size(in,1)*0.7),:);

out_training=out(1:round(size(in,1)*0.7),:);

in_test=in(round(size(in,1)*0.7)+1:end,:);

out_test=out(round(size(in,1)*0.7)+1:end,:);

t = templateTree('Surrogate','On');

%load('new_data_size10.mat')

bc=fitrensemble(in_training,out_training,'Method','LSBoost');

%bc=fitrensemble(in_training,out_training,'AdaBoostM1');

%bc=fitrensemble(in_training,out_training,'OptimizeHyperparameters','auto','Learners',t, ...

% 'HyperparameterOptimizationOptions',struct('AcquisitionFunctionName','expected-improvement-plus'))

pre_train=bc.predict(in_training);

pre_tast=bc.predict(in_test);

MAE_Training=mean(abs(out_training-pre_train));

MSE_Training=mean(abs(out_training-pre_train).^2);

MAE_Test=mean(abs(out_test-pre_tast));

MSE_Test=mean(abs(out_test-pre_tast).^2);

disp('--------------------------------');

disp(['MAE Training = ',num2str(MAE_Training)])

disp(['MAE Test = ',num2str(MAE_Test)])

disp('-------------Training-------------------');

p_training=CalcPerf(out_training,bc.predict(in_training))

disp('-------------Test-------------------');

p_test=CalcPerf(out_test,bc.predict(in_test))

if(MAE_Training<MAE_Test && MAE_Test<0.0105)

break

end

end

1. A sample of Matlab Codes written to illustrate 3D plots in the study. Code is related to 3D plots in Figure 2.

clc, clear, close all;

%warning('off','all')

%warning

load('data11.mat')

n=randperm(size(in,1));

in=in(n,:);

out=out(n);

in_training=in(1:round(size(in,1)*0.7),:);

out_training=out(1:round(size(in,1)*0.7),:);

in_test=in(round(size(in,1)*0.7)+1:end,:);

out_test=out(round(size(in,1)*0.7)+1:end,:);

t = templateTree('Surrogate','On');

load('new_data_size11.mat')

bc=fitrensemble(in_training,out_training,'Method','LSBoost');

%bc=fitrensemble(in_training,out_training,'AdaBoostM1');

%bc=fitrensemble(in_training,out_training,'OptimizeHyperparameters','auto','Learners',t, ...

% 'HyperparameterOptimizationOptions',struct('AcquisitionFunctionName','expected-improvement-plus'))

pre_train=bc.predict(in_training);

pre_tast=bc.predict(in_test);

MAE_Training=mean(abs(out_training-pre_train));

MSE_Training=mean(abs(out_training-pre_train).^2);

MAE_Test=mean(abs(out_test-pre_tast));

MSE_Test=mean(abs(out_test-pre_tast).^2);

disp('--------------------------------');

disp(['MAE Training = ',num2str(MAE_Training)])

disp(['MAE Test = ',num2str(MAE_Test)])

disp('-------------Training-------------------');

p_training=CalcPerf(out_training,bc.predict(in_training))

disp('-------------Test-------------------');

p_test=CalcPerf(out_test,bc.predict(in_test))

arr35=zeros(46,39);

a=15:60;

b=27:65;

for i=1:46

for j=1:39

arr35(i,j)=bc.predict([35,a(i),b(j)]);

end

end

[x,y]=meshgrid(27:65,15:60);

gca11_35=surf(x,y,arr35);

xlabel('Time (m)')

ylabel('Temp (°C)')

zlabel('PDI')

saveas(gca11_35,'PDI_35.emf');

arr40=zeros(46,39);

for i=1:46

for j=1:39

arr40(i,j)=bc.predict([40,a(i),b(j)]);

end

end

[x,y]=meshgrid(27:65,15:60);

gca11_40=surf(x,y,arr40);

xlabel('Time (m)')

ylabel('Temp (°C)')

zlabel('PDI')

saveas(gca11_40,'PDI_40.emf');

1. Chen X, Zou LQ, Niu J, Liu W, Peng SF, Liu CM. The Stability, Sustained Release and Cellular Antioxidant Activity of Curcumin Nanoliposomes. Molecules. 2015;20(8):14293-311. Epub 2015/08/08. doi: 10.3390/molecules200814293. PubMed PMID: 26251892; PubMed Central PMCID: PMCPMC6331986.
